# Models and Castings

**Published:** 1857-06

**Authors:** 


### MODELS AND CASTINGS.

After eleven year's experience in mechanical dentistry, and having seen many *labored* attempts in dental periodicals, at instructions in its various departments; allow me to say a few words in relation to a method of getting up castings, practiced by myself and other dentists, to whom I have communicated the plan.

Premising that every *dentist* knows how to take an impression and fill it with plaster, we will commence operations with the *plaster model.*

This should be made to set perfectly *level*, so as not to tilt in moulding; this is easily done, by turning plaster on to a flat surface of glass, stone, or porcelain, and setting the model into it; at the same time another advantage is gained, of placing a model that is *under-cut*, in such a position that it will draw readily from the sand; (a little care in this matter will enable one to make a very bad model draw almost perfect.)

After trimming the sides *smooth*, and arranging *chamber* and *varnishing*, let the surface get well dried, and there will be no need of *charcoal* dust upon it.

*Sand* for castings never should be *wet;* instead of water, use *sweet-oil*, and when *once* moistened, it will *always* be ready for use. One quart of oil will be sufficient for about two pecks of sand; should the sand be too moist, it will bubble, but add a little more sand, and no farther trouble will be experienced.

Any one who adopts this method of preparing sand, will never relinquish it.

In moulding, pack the sand *on to the model* and around it, as solid as possible with a stick, and deliver it by jarring the ring, as a model will always *deliver itself*, better than it can be picked out.

For *metal*, use what is called babbit, or anti-friction metal, for the following reasons, viz:

1st. There is *no shrinkage* to it.

2d. It is *harder* than any other metal used for this purpose, except iron or brass.

3d. It melts at a low temperature, consequently can be turned into the sand so cool, as not to burn it. I can safely affirm that it is the *very best metal* for this purpose that can be used.

It can be obtained in most of the principal cities north and west, at from 40 to 50 cents per pound, and if not allowed to get *very hot* will not oxydize, and will not prove an expensive metal, as some might suppose.

When it cannot be bought near at hand, it can readily be made as follows:

Copper, 1 part.
Metallic Antimony, 2 parts.
Tin, 6 parts.

Fuse, in a black lead, or common crucible, *in the order named.* Should it be brittle, add a little more tin.

Before turning the metal into the mould, stir well till it *begins* to thicken, and never cool it by putting into cold water, while it is very hot, as it would become brittle.

Wash the surface with clay, mixed with alcohol, as it dries sooner than water. Imbed the casting in sand as deep as necessary, and place the ring around it.

For female cast use lead, to which add tin, one-sixth or one eighth, this makes it melt at a lower temperature, so that ti will not stick to the babbit metal, but it should be turned as *cold as possible.* Lead hardened with tin, as a "*bed-piece*," will strike up a plate better than lead alone, as it is *too* soft.

Hoping some dentist may find his troubles and perplexities somewhat abated by the adoption of this method, and begging pardon for occupying so much space, I will trouble you no farther. L. P. H.

MILWAUKEE, March 1857.

